# Nuclear Lamin B1 Interactions With Chromatin During the Circadian Cycle Are Uncoupled From Periodic Gene Expression

**DOI:** 10.3389/fgene.2019.00917

**Published:** 2019-10-03

**Authors:** Annaël Brunet, Frida Forsberg, Qiong Fan, Thomas Sæther, Philippe Collas

**Affiliations:** ^1^Department of Molecular Medicine, Institute of Basic Medical Sciences, Faculty of Medicine, University of Oslo, Oslo, Norway; ^2^Department of Immunology and Transfusion Medicine, Oslo University Hospital, Oslo, Norway

**Keywords:** circadian rhythm, lamin B, lamina-associated domain, oscillation, period

## Abstract

Many mammalian genes exhibit circadian expression patterns concordant with periodic binding of transcription factors, chromatin modifications, and chromosomal interactions. Here we investigate whether chromatin periodically associates with nuclear lamins. Entrainment of the circadian clock is accompanied, in mouse liver, by a net gain of lamin B1–chromatin interactions genome-wide, after which the majority of lamina-associated domains (LADs) are conserved during the circadian cycle. By tailoring a bioinformatics pipeline designed to identify periodic gene expression patterns, we also observe hundreds of variable lamin B1–chromatin interactions among which oscillations occur at 64 LADs, affecting one or both LAD extremities or entire LADs. Only a small subset of these oscillations however exhibit highly significant 12, 18, 24, or 30 h periodicity. These periodic LADs display oscillation asynchrony between their 5′ and 3′ borders, and are uncoupled from periodic gene expression within or in the vicinity of these LADs. Periodic gene expression is also unrelated to variations in gene-to-nearest LAD distances detected during the circadian cycle. Accordingly, periodic genes, including central clock-control genes, are located megabases away from LADs throughout circadian time, suggesting stable residence in a transcriptionally permissive chromatin environment. We conclude that periodic LADs are not a dominant feature of variable lamin B1–chromatin interactions during the circadian cycle in mouse liver. Our results also suggest that periodic hepatic gene expression is not regulated by rhythmic chromatin associations with the nuclear lamina.

## Introduction

Thousands of mammalian genes exhibit autonomous oscillatory patterns of expression concordant with the circadian (24 h) rhythm (Hastings et al., 2018). The circadian rhythm is governed by central and peripheral clocks, respectively in the nervous system and in individual organs including adipose tissue, lungs and liver, controlled by transcriptional and translational negative feedback loops (Takahashi, 2017). The core clock is regulated by the CLOCK and BMAL1 transcription factors (TFs) which drive expression of clock-controlled genes including *Per*, *Cry*, *Nr1d1/Nr1d2* (encoding REV-ERB alpha/beta proteins, respectively), and *Ror* genes (encoding ROR alpha/beta/gamma), by binding to E-boxes in their promoters. The PER-CRY repressor complex inhibits activity of CLOCK–BMAL1, lowering transcription of *Per* and *Cry* and generating a negative feedback loop. RORs and REV-ERBs act as activators and repressors, respectively, of *Arntl* (also called *Bmal1*) and other clock genes, driving their rhythmic transcription. Stability of PER and CRY proteins is regulated by post-translational modifications leading to their time-dependent degradation, enabling a new cycle of CLOCK–BMAL1-driven gene expression.

Circadian binding of TFs and chromatin modifiers to promoters and enhancers generates rhythmic chromatin modifications and remodeling (Koike et al., 2012; Masri et al., 2014; Zhang et al., 2015; Kim et al., 2018). In mouse liver, histone H3 lysine 4 trimethylation (H3K4me3) levels oscillate at promoters of circadian genes (Vollmers et al., 2012; Aguilar-Arnal et al., 2015), while rhythmic H3K4me1 and H3K27 acetylation (H3K27ac) levels define oscillating enhancers (Koike et al., 2012; Vollmers et al., 2012; Fang et al., 2014; Takahashi, 2017). Recruitment to chromatin of the sirtuin SIRT1, a histone deacetylase (HDAC) involved in circadian control of metabolism (Nakahata et al., 2008; Masri et al., 2014), is under influence of oscillatory levels of metabolites (Aguilar-Arnal et al., 2015) and provides a molecular link between metabolism, chromatin and circadian rhythms. Periodic recruitment of HDAC3 to chromatin also regulates circadian rhythms (Feng et al., 2011). These oscillatory cistromes and chromatin modifications raise the possibility that other chromatin-linked processes also show rhythmic patterns. Indeed, periodic promoter–enhancer interactions regulate and connect circadian liver gene expression networks (Aguilar-Arnal et al., 2013; Xu et al., 2016; Kim et al., 2018; Mermet et al., 2018). Thus, circadian-dependent changes in chromatin topology contribute to shaping the nuclear landscape (Yeung and Naef, 2018).

Dynamic interactions of chromatin with the nuclear lamina, a meshwork of A-type lamins [lamins A and C (LMNA and LMNC)], products of the *Lmna* gene, and B-type lamins [lamins B1 and B2 (LMNB1 and LMNB2)], encoded by the *Lmnb1* and *Lmnb2* genes respectively, at the nuclear periphery (Burke and Stewart, 2013) also constitute one mechanism of regulation of gene expression (van Steensel and Belmont, 2017). Interestingly, A- and B-type lamins are not only found at the nuclear periphery, where the nuclear lamina is located, but also in the nucleoplasm where interactions with chromatin have been reported to also occur (Naetar et al., 2017; Pascual-Reguant et al., 2018). Regions of chromatin interacting with lamins, so-called lamina-associated domains (LADs), are typically heterochromatic and relatively well conserved between cell types (Peric-Hupkes et al., 2010). However, other LADs are variable and altered during differentiation (Peric-Hupkes et al., 2010; Rønningen et al., 2015; Poleshko et al., 2017; Paulsen et al., 2019). It remains however unclear to what extent variable LADs arise and disappear as a consequence of regulatory mechanisms or through random interactions of chromatin with nuclear lamins. Whether individual loci or broader domains such as LADs display oscillatory interactions with nuclear lamins has also to our knowledge not been addressed.

Scarce evidence links the nuclear envelope to circadian gene expression. HDAC3, a component of the clock negative feedback loop (Shi et al., 2016) and a regulator of lamina-associated genes (Demmerle et al., 2013), interacts with the inner nuclear membrane proteins TMPO/lamina-associated polypeptide 2β (Somech et al., 2005) and emerin (Demmerle et al., 2013). The clock regulators SIRT1 and SIRT6 deacetylases interact with LMNA (Liu et al., 2012; Ghosh et al., 2015) at the nuclear lamina, where they modulate histone acetylation and gene expression. *In vitro*, BMAL1 expression seems to be modulated by MAN1, another protein of the inner nuclear membrane, through MAN1 binding to the *ARNTL* (also called *BMAL1*) promoter (Lin et al., 2014). Lastly, in a human colon cancer cell line, a handful of circadian genes have been shown to rhythmically interact with the nuclear lamina, regulating their transcription (Zhao et al., 2015). These observations suggest that nuclear lamins may contribute to the regulation of circadian gene expression. However, whether chromatin exhibits genome-scale periodic associations with the lamina has not been examined.

Here, we determined whether chromatin exhibits periodic interactions with LMNB1 after entrainment of the circadian clock in mouse liver. We opted to examine this feature of genome organization in the liver because it is highly responsive to entrainment of the circadian clock at the metabolic level and as such is the most studied organ in investigations of circadian control of transcriptional regulation (Takahashi, 2017) and spatial chromatin conformation (Aguilar-Arnal et al., 2013; Kim et al., 2018). We show that periodic lamin B1–chromatin interactions are not a dominant feature of LADs during the circadian cycle and are uncoupled from periodic gene expression. Our data strongly suggest that periodic gene expression is not under direct regulation of rhythmic association of chromatin with the nuclear lamina.

## Materials and Methods

### Mice

Wild-type C57Bl/6 male mice (Jackson Laboratories) were housed in 12 h light/12 h dark cycles with lights on at 6 am and lights off at 6 pm. Mice were kept off chow for 24 h, refed *ad libitum* at circadian time CT0 (6 am) and sacrificed at CT6, 12, 18, 24, and 30 h (n = 7 mice per CT). Non-synchronized (NS) mice (n = 7) were sacrificed at 12:00 noon on the day prior to food restriction. Livers were collected from all mice, partitioned and snap-frozen in liquid nitrogen. Procedures were approved by the University of Oslo and Norwegian Regulatory Authorities (approval No. 8565).

### RNA-Sequencing and Gene Expression Analysis

Total RNA was isolated from livers of five mice at each CT using the RNeasy Mini Kit (Qiagen). RNA (1 μg) was reverse-transcribed (BioRad Laboratories) and analyzed by qPCR using IQ SYBR green (BioRad Laboratories), *Eif2a* as reference and primers listed in **Supplementary Table S1** (n = 5 mice per CT). RNA was also processed to prepare RNA-sequencing (RNA-seq) libraries (TruSeq Stranded mRNA Library Prep Kit; Illumina; n = 3 mice per CT) which were sequenced on an Illumina HiSeq2500. RNA-seq reads were processed with Tuxedo (Trapnell et al., 2010). TopHat v2.10 was used to align reads with no mismatch against the mm10 genome (Langmead and Salzberg, 2012). Transcript level was estimated using cufflinks v2.2.1 and differential gene expression determined using cuffdiff v2.2.1 (Trapnell et al., 2010). Gene expression plots show mean ± SD relative expression levels (for RT-qPCR data) or FPKM (fragments per kilobase of exon model per million reads mapped; RNA-seq data) at each CT, as indicated, with single data points. A gene was ascribed to a LAD if its transcription start site overlapped with the LAD.

### MetaCycle Analysis

We used MetaCycle (Wu et al., 2016) to identify genes with periodic expression patterns. MetaCycle measures the goodness-of-fit between RNA-seq, FPKM, and theoretical cosine curves with varying periods and phases. The extrapolated periodic function best fitting the RNA-seq data is selected and the significance of a given periodicity is determined by assigning *P* values after scrambling FPKM values. MetaCycle was applied to the entire range of CTs in the study (30 h). To fit RNA-seq data with periodic functions, MetaCycle normalizes FPKM values by computing z-scores. Our time series data are integer intervals with even sampling and do not include missing values. Given the features of our time series data, MetaCycle incorporated both the JTK_CYCLE (JTK) and the Lomb-Scargle (LS) methods for periodic signal detection. Based on our data and time resolution, available period values are integers (0, 6, 12, 18, 24, 30, and 36 h) for JTK and real numbers ranging from 12 to 48 h for LS. Periods are the mean of JTK and LS period values. Thus we focused our analyses on oscillations with 12, 18, 24, and 30 h periods, each ± 3 h, i.e. half the time resolution in our study. Moreover, the restricted 12 ± 3 h period group was not able to distinguish groups of 12 h periodic genes oscillating in positive (Φ_π/2) or negative (Φ_−π/2) quadrature of phase, but only those oscillating in phase or opposition of phase. MetaCycle was also applied to identify periodicity in LAD coverage (see below).

### Liver Extracts and Immunoblotting

Mouse liver samples were homogenized in ice-cold phosphate buffered saline (PBS) with 1× protease inhibitor cocktail (Sigma-Aldrich) and 1 mM phenylmethylsulfonide fluoride (PMSF; Sigma-Aldrich), using a Dounce tissue grinder with an A-type glass pestle, followed by centrifugation at 200 g for 5 min at 4°C. Supernatants were discarded, and pellets washed once in ice-cold PBS with 1× protease inhibitor cocktail and 1 mM PMSF and centrifuged at 200 g for 5 min at 4°C. Cells were lysed in RIPA buffer (140 mM NaCl, 10 mM Tris–HCl pH 8.0, 1 mM EDTA, 0.5 mM EGTA, 1% Triton X-100, 0.1% SDS, 0.1% sodium deoxycholate, 1 mM PMSF, protease inhibitors) with 1× protease inhibitor cocktail and 1 mM PMSF, sonicated twice with 30 s on/off in a Bioruptor (Diagenode), and incubated under rotation at 25 rpm for 30 min at 4°C. Lysates were centrifuged at 10,000 g for 10 min at 4°C and supernatants collected for immunoblotting analysis. Proteins were separated by 7.5% SDS-PAGE, transferred onto an Immobilon-FL membrane (Millipore) and membranes blocked with 3% BSA in TBST (0.15 M NaCl, 50 mM Tris–HCl pH 7.6, 0.05% Tween^®^20). Membranes were incubated with antibodies against CLOCK (1:500; Abcam ab3517), LMNB1 (1:1,000; Santa Cruz Biotechnology sc6216), and β-actin (1:2,000; Sigma-Aldrich A5441) in TBST with 3% BSA. Secondary horseradish peroxidase-conjugated anti-mouse (Jackson ImmunoResearch #115-035-174) and anti-goat (Rockland #605-4302) antibodies were used at 1:10,000 dilutions in TBST with 3% BSA.

### Chromatin Immunoprecipitation (ChIP)

ChIP of LMNB1 was done as described earlier (Rønningen et al., 2015) and adapted for liver pieces. Snap-frozen liver tissue pieces (40–50 mg, in liquid nitrogen) were thawed on ice and minced on ice for 30 s. Minced tissue was resuspended in PBS containing 1 mM PMSF and protease inhibitors, and homogenized by 7–10 strokes in a 2-ml Dounce homogenizer using a pestle B (tight-fitting). Samples were centrifuged at 400 g and supernatants discarded. Pellets were resuspended in PBS containing 1% formaldehyde (Sigma-Aldrich) and cross-linking was allowed to occur for 10 min at room temperature. Cross-linked samples were sedimented and lysed in RIPA buffer. Chromatin was fragmented by sonication (4 times 10 min) in a Bioruptor (Diagenode). After sedimentation, the supernatant was diluted 10-fold in RIPA buffer and incubated with anti-LMNB1 antibodies (10 µg; Abcam ab16048) coupled to Dynabeads Protein G (Invitrogen) overnight at 4 °C. ChIP samples were washed 3 times in ice-cold RIPA buffer. Cross-links were reversed and DNA eluted for 6 h at 68°C in 50 mM NaCl, 20 mM Tris–HCl pH 7.5, 5 mM EDTA, 1% SDS and 50 ng/μl proteinase K. DNA was purified by phenol-chloroform-isoamylalcohol extraction and used for qPCR (see **Supplementary Table S1** for primer information) or to prepare libraries (Illumina) for sequencing on an Illumina HiSeq2500. Input ChIP DNA consisted of fragmented chromatin aliquots (as above) incubated overnight at 4 °C with no antibodies or beads and subsequently processed as, and in parallel with, the ChIP samples.

### LMNB1 ChIP-Seq Data Processing

LMNB1 ChIP-seq and input reads were mapped to the mm10 genome using Bowtie v2.25.0 (Langmead et al., 2009) with default parameters after removing duplicates using Picard’s MarkDuplicates. To avoid normalization bias, we ensured that each pair of mapped ChIP and input read files had the same read depth by down-sampling reads for each chromosome individually. Mapped reads were used to call LADs using Enriched Domain Detector (EDD) (Lund et al., 2014) with the following alterations (Forsberg et al., 2019) as described here. To account for technical variation occurring in LAD calling, we first ran EDD 10 times on each LMNB1 ChIP-seq dataset in auto-estimation mode for GapPenalty (GP) and BinSize (BS). Average GP standard deviation was ≤ 1.6 units while BS did not vary (**Supplementary Table S2**). GP variations elicited minimal alterations in LAD calls, allowing estimation of technical variability. For all LADs, median length of these variations was 0.32 Mb; this is < 1% of total LAD coverage, and 3–15 times smaller than median LAD sizes for each CT and replicate. Thus intrinsic EDD variability did not significantly impact LAD calling. Average GP and BS values from the 10 runs were used to set GP and BS before a final EDD run with each ChIP-seq dataset (**Supplementary Table S2**). Intersects between LADs and genes were determined using BEDTools v2.21.0 (Quinlan and Hall, 2010) and BEDOPS v2.4.27 (Neph et al., 2012). Scripts were written in Bash, Perl, or R and ggplot2 in R was used for plots. Browser files were generated by calculating ChIP/input ratios in 10-kb bins with input normalized to the ratio of total ChIP reads over total input reads.

### Determination of Periodic Lads With MetaCycle

To quantify the genomic coverage of variation in the length of LADs during the circadian cycle, we determined for each LAD its “maximal coverage” (cov_max_) as the union of LAD coverage across all replicates and CTs, and attributed to this cov_max_ a reference value of 1. The cov_max_ 5′ and 3′ limits provided genomic coordinates for measures of variations in LAD length within cov_max_. For each CT and replicate, variable 5′ and 3′ genomic lengths (in base pairs) were extracted and standardized from 0 to 1, 0 referring to the complete disappearance of a LAD and 1 being the cov_max_ value of the LAD. MetaCycle was applied to determine the periodicity of LAD extensions and shortenings at the 5′ and 3′ borders. Period groups were defined as for RNA-seq analysis (12, 18, 24, and 30 h, each ± 3 h). The method is described in more detail as part of the *Results* section.

### Randomization to Validate Periodic LAD Significance

A randomization test was done as additional validation of extrapolated MetaCycle LAD periodicity. To this end, we shuffled the measured experimental variations in 5′ and 3′ LAD lengths within the cov_max_ area for all datasets across CTs and replicates; this was done 3 times. MetaCycle was applied to each randomized order of CTs to identify any periodicity in the variations in LAD lengths across these randomized CTs. The extrapolated periodicity given by MetaCycle was compared with the periodicity found for the experimental order of CTs. If experimental LAD periodicity was different from at least two randomized CTs, it was considered as imposed by the order of CT and not due to random lamin–chromatin interactions.

### Determination of Gene-LAD Distance

Gene-to-nearest LAD distance was calculated as the distance from the 5′ and 3′ end of a gene to, respectively, the nearest 3′ and 5′ LAD border. Gene strand was respected and LAD intersects were used at each CT. If a gene was entirely in a LAD, gene-to-nearest LAD distance was the distance from the 5′ and 3′ end of the gene to the first neighboring LAD, both upstream and downstream. In our approach, internal LAD configuration within the cov_max_ area, such as a LAD split or fusion, was not considered in the determination of periodicity at the 5′ and 3′ borders.

### Data Viewing

Genome browser views were produced with the Integrated Genomics Viewer (Robinson et al., 2011) using the mm10 genome annotation to illustrate LADs in regions of interest.

### Statistics

MetaCycle used its built-in statistical method to assign *P* values from Fisher’s exact tests. The JTK and LS methods used in MetaCycle assign a *P* value for each fitted data type, i.e. gene expression variation for RNA-seq and RT-qPCR, protein expression level for Western blot quantification or LAD size variation for LMNB1 ChIP-seq data. These multiple *P* values are combined into a one-test Chi-square statistics assuming a Chi-squared distribution with 2 k degrees of freedom (where k is the number of *P* values; here k = 3 for RNA-seq data, k = 5 for RT-qPCR, k = 3 for Western blot data and k = 2 for LAD data), when all null-hypotheses are true and each *P* value is independent. The combined *P* value was determined by the Chi-square *P* value and was used to determine the significance of oscillating patterns. Protein levels in Western blots were compared pair-wise between time-points using paired t-tests generating two-tailed *P* values; P < 0.01 was considered significant.

## Results

### Hepatic Genes Exhibit Distinct Periodic Transcript Levels in Liver

**Figure 1:**
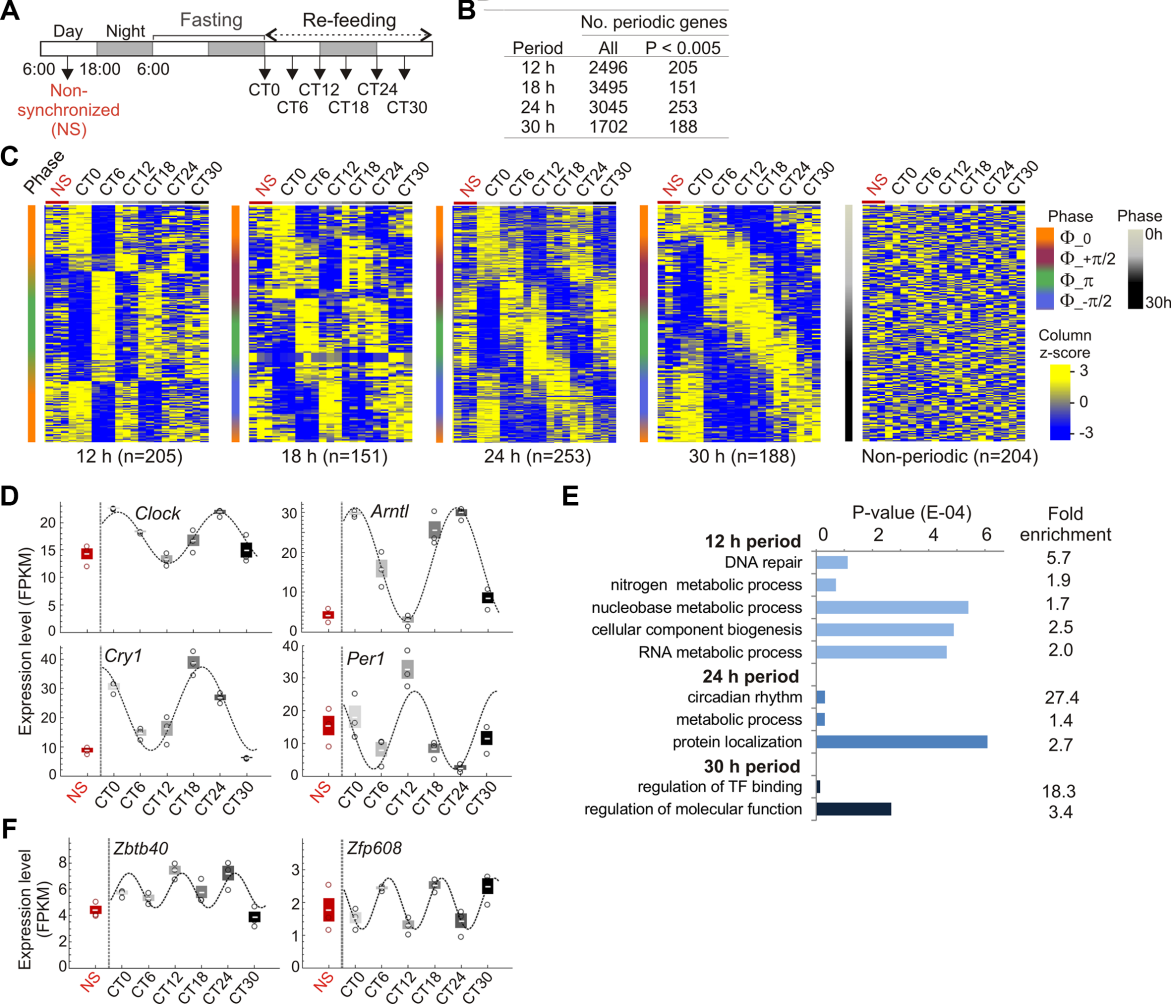
Fasting and refeeding entrains circadian gene expression in liver. **(A)** Circadian clock entrainment schedule used in this study. **(B)** Numbers of periodic genes. **(C)** Expression profiles (FPKM z-scores) of periodic and non-periodic genes in non-synchronized (NS) mice and from CT0 to CT30 (3 mice per CT). Genes are ranked by increasing phase value (y axis; scale on the right). **(D)** RNA-seq analysis of circadian expression patterns of central clock control genes (mean ± SD, individual values). **(E)** Enriched GO terms for periodic genes. **(F)** Circadian expression patterns of the BTB/POZ domain transcription factor gene *Zbtb40* and of the zinc-finger transcription factor gene *Zfp608* (FPKM z-scores; mean ± SD, individual values). In **(D)** and **(F)** cosine curves are MetaCycle best-fits; see **Supplementary Table S3** for details.

To entrain the circadian clock, mice were subjected to 24 h fasting and refed *ad libitum* from circadian time CT0 (Tahara et al., 2011). Livers were collected every 6 h until CT30, and from non-synchronized (NS) mice 18 h before the fasting period (**Figure 1A**). Entrainment of the clock was confirmed by periodic expression of the core clock genes *Clock*, *Arntl* (*Bmal1*), *Cry1*, *Per1*, and *Nr1d1*, assessed by RT-qPCR and analysis of their periodicity using MetaCycle (**Supplementary Figure 1**; **Supplementary Table S3**). We used MetaCycle to identify genes with periodic expression patterns from RNA-seq data generated in biological triplicates at each CT. MetaCycle measures the goodness-of-fit between RNA-seq FPKM and cosine curves with varying periods and phases. The extrapolated periodic function best fitting the data is selected and significance is determined (Fisher’s exact tests) after scrambling FPKM values. From the period distribution for all 17,330 expressed genes in liver (expressed at least at one time point), we focused on oscillations with 12, 18, 24, and 30 h periods (each ± 3 h, half the time resolution in our study) (**Supplementary Figure S2A**). We find that nearly 20% of oscillations are circadian (24 h period; 3,046 genes), and thousands of genes oscillate with periods within the circadian rhythm and beyond (**Figure 1B**), in line with previous reports (Hughes et al., 2009; Korencic et al., 2014; Zhu et al., 2017). Among these, a subset displays highly significantly oscillating transcripts (*P* < 0.005; **Figures 1B and C**). Within a given period, mRNA oscillations occurred with distinct phases (**Supplementary Figure S2B**): oscillations are in phase (Φ = 0, time at first maxima) and opposition of phase (Φ = π), and for 18, 24, and 30 h periods, are also offset by one-quarter cycle (Φ = ½ π and Φ = −½ π) (**Figure 1C**; **Supplementary Figures S2C and D**). Significantly periodic genes include the core clock genes (**Figure 1D**; **Supplementary Figure S2E**), confirming entrainment of the clock. Genes (644) also display significant (*P* < 0.005) mRNA oscillations with periods outside 12, 18, 24, or 30 h. We also defined a set of 204 “non-periodic” genes with mRNA levels discordant (1 > *P* > 0.9999) with any cosine curve tentatively fitted by MetaCycle (**Figure 1B**). Lists of periodic and non-periodic genes are provided in **Supplementary Tables S4** and **S5**.

Gene ontology analysis confirms enrichment of 24 h periodic genes in rhythmic and circadian processes, including key metabolic functions (**Figure 1E**; **Supplementary Table S6**). A number of periodic genes encode BTB/POZ domain TFs, some of which are involved in targeting chromatin to the nuclear lamina (Zullo et al., 2012) (**Supplementary Table S4**). Some of these genes are CLOCK or BMAL1 targets (e.g. *Zbtb40*, *Zbtb7b/cKrox*, and *Zfp608*; **Figure 1F**) and could tentatively be involved in associations of chromatin with the nuclear envelope.

### Entrainment of the Circadian Clock Resets LMNB1–Chromatin Interactions

**Figure 2:**
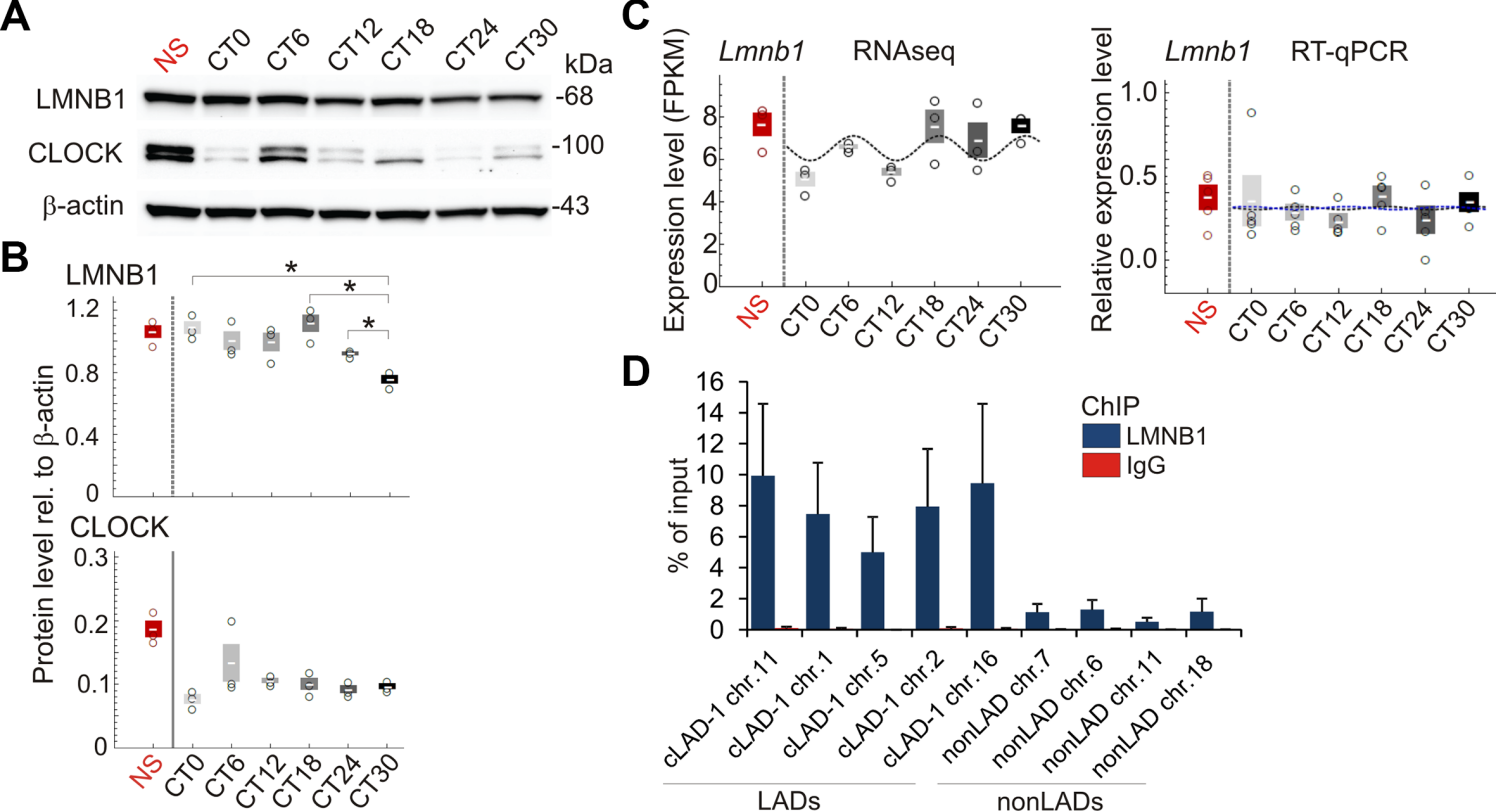
Expression of LMNB1 protein and transcripts during the circadian cycle. **(A)** Western blots of LMNB1 and CLOCK expression; β-actin was used as loading control; see **Supplementary Figure S3A** for all biological replicates. **(B)** Quantification of Western blots from biological triplicates, relative to β-actin; mean ± SD, individual values; *P < 0.01 (paired t-tests, two tailed *P* values). Note the difference in variation of expression of CLOCK protein and *Clock* transcripts expected from the regulation of protein expression at the transcriptional and protein degradation levels). **(C)** Levels of *Lmnb1* transcripts determined by RNA-seq (FPKM; n = 3) and RT-qPCR (n = 5; expression relative to *Eif2a* levels); means ± SD, individual values. Cosine curves are MetaCycle best-fits. *Lmnb1* is not significantly periodic (RNA-seq *P* = 0.16; RT-qPCR *P* = 0.98; MetaCycle Fisher’s exact tests). RT-qPCR graph: blue line, MetaCycle best-fit from RT-qPCR data; black line, MetaCycle best-fit from RNA-seq data; note the overlap between the two, indicating strong concordance between the two datasets. Amplitude and base values used for both fits are from RT-qPCR MetaCycle analysis. **(D)** ChIP-qPCR analysis of LMNB1 occupancy in constitutive LADs and in non-LAD regions (mean ± SD of % of input chromatin analyzed by qPCR; n = 3 NS mice); ChIP using an irrelevant IgG was done as negative control.

We therefore examined chromatin association with nuclear lamins in liver during the circadian cycle. We first established that LMNB1 protein levels vary moderately over time but do not significantly oscillate (*P* = 0.61; Fisher’s exact test), and that *Lmnb1* transcripts analyzed by RNA-seq and verified by RT-qPCR do not periodically oscillate (**Figures 2A–C**; **Supplementary Figure S3A**). Moreover, chromatin immunoprecipitation (ChIP)-qPCR of LMNB1 from liver confirmed LMNB1 enrichment in LADs found in several other mouse cell types (constitutive LADs; cLADs) (Meuleman et al., 2013) (**Figure 2D**).

**Figure 3:**
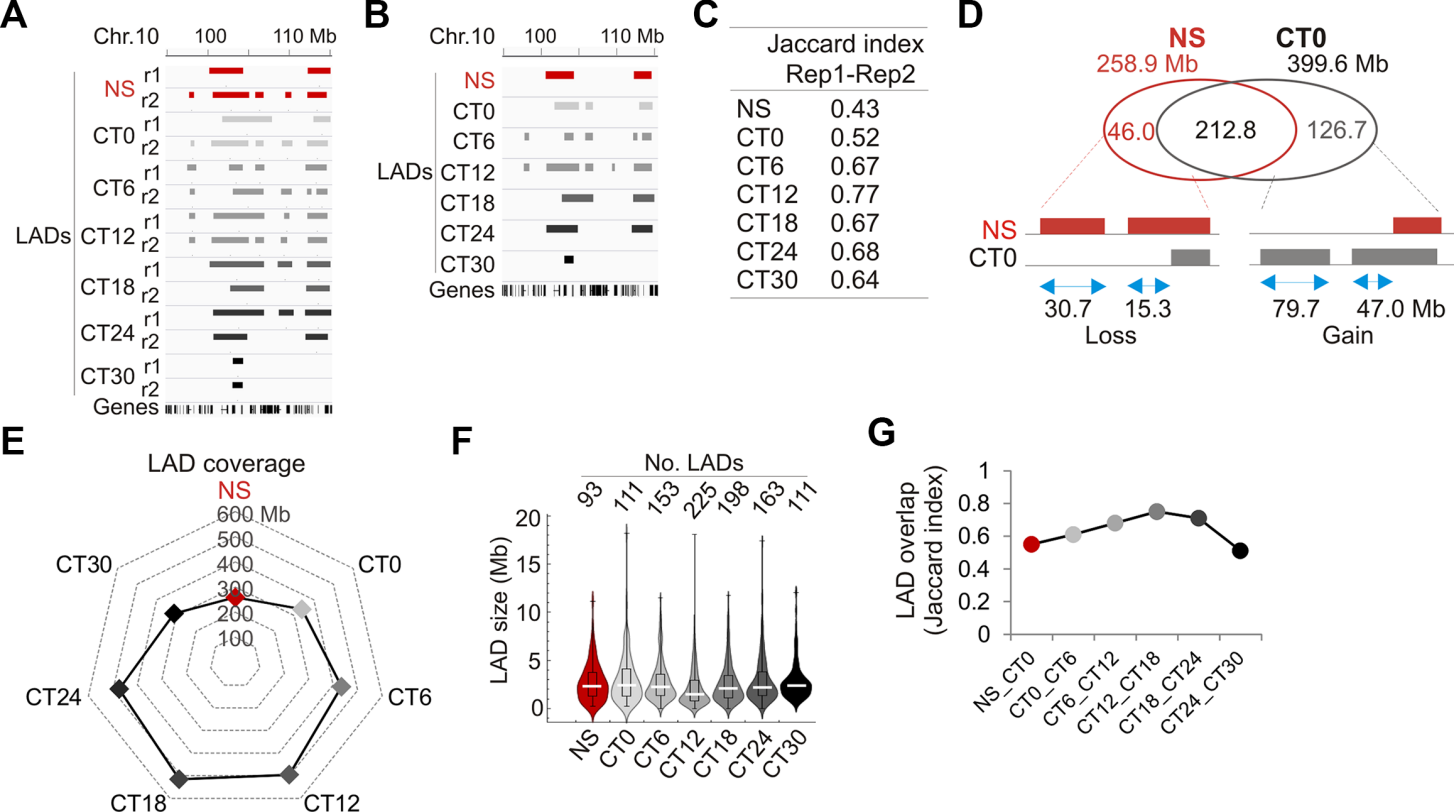
Characterization of LADs during the circadian cycle. **(A)** Genome browser view of LADs in biological replicate (r1, r2) and at each CT for the indicated region on chromosome 10. **(B)** Genome browser view of LAD intersects between replicates for each CT in the same region as in **(A)**. **(C)** Jaccard indices of LAD overlap between replicates. **(D)** Venn diagram of LAD overlap between NS mice and CT0 (intersect of replicates); bottom, schematic representation of genome coverage by LADs gained or lost at CT0 relative to NS, for stand-alone LADs and LAD extension or shortening. **(E)** Radar plot of genome coverage by LADs. **(F)** Distribution and median of LAD size. **(G)** Jaccard indices of LAD overlap between consecutive CTs.

Thus, we determined to what extent LADs varied during the circadian cycle. We performed a ChIP-seq analysis of LMNB1 from livers of NS mice and at each CT in biological replicates (i.e. two mice per CT) and mapped LADs (**Figure 3A**; see **Supplementary Table S2**). We find that LAD sizes are overall constant and LADs display low gene density (**Supplementary Figures S3B and C**). LADs also show robust overlap between replicates (**Figure 3A and B**), as shown by Jaccard indices (**Figure 3C**) and by minimal variations between intersect coverage and replicates (< 1 Mb, or 0.05–0.21% of LAD coverage per replicate; **Supplementary Table S7**). Thus unless specified otherwise, LADs were subsequently analyzed at each time point as intersects between replicates.

We next assessed to what extent entrainment of the circadian clock did reset LADs. We find that entrainment of the clock is manifested by a LAD gain of 126.7 Mb at CT0 relative to NS, as stand-alone LADs or as extensions of existing LADs (**Figure 3D**). This increase in LMNB1–chromatin interactions was confirmed by ChIP-qPCR (**Supplementary Figures S3D and E**). We also note a LAD loss of 46 Mb at CT0 relative to NS, mostly as stand-alone LADs (**Figure 3D**). Thus, entrainment of the clock is associated with a net gain of LMNB1–chromatin interactions.

Comparison of LAD coverage over time reveals an increase from CT0 (339 Mb) to CT12 (496 Mb), followed by a decrease to CT30 (312 Mb) (**Figure 3E**), likely resulting from variations in LAD numbers rather than LAD size (**Figure 3F**). LADs nevertheless display robust overlap across CTs (**Figure 3G**; **Supplementary Figure S3F**), indicating that they are overall conserved in liver during the circadian cycle. However, the data also indicate that some changes in LMNB1–chromatin interactions do occur, and underline the detection of variable LADs. Of note, although LMMB1 is predominantly found at the nuclear lamina at the nuclear periphery, a fraction of LMNB1 has also been reported in the nucleoplasm, in association with nucleoli in heterochromatin domains (Sen Gupta and Sengupta, 2017) and with domains of euchromatin (Pascual-Reguant et al., 2018). Moreover, our ChIP approach to identify LMNB1 LADs does not *a priori* discriminate between the nuclear peripheral and internal pools of LMNB1. Thus we do not at present exclude that a subset of LMNB1–chromatin interactions detected during the circadian cycle in our study potentially involves a nucleoplasmic LMNB1 pool.

### Periodic Oscillations in LMNB1–Chromatin Interactions Constitute a Minor Proportion of LADs

**Figure 4:**
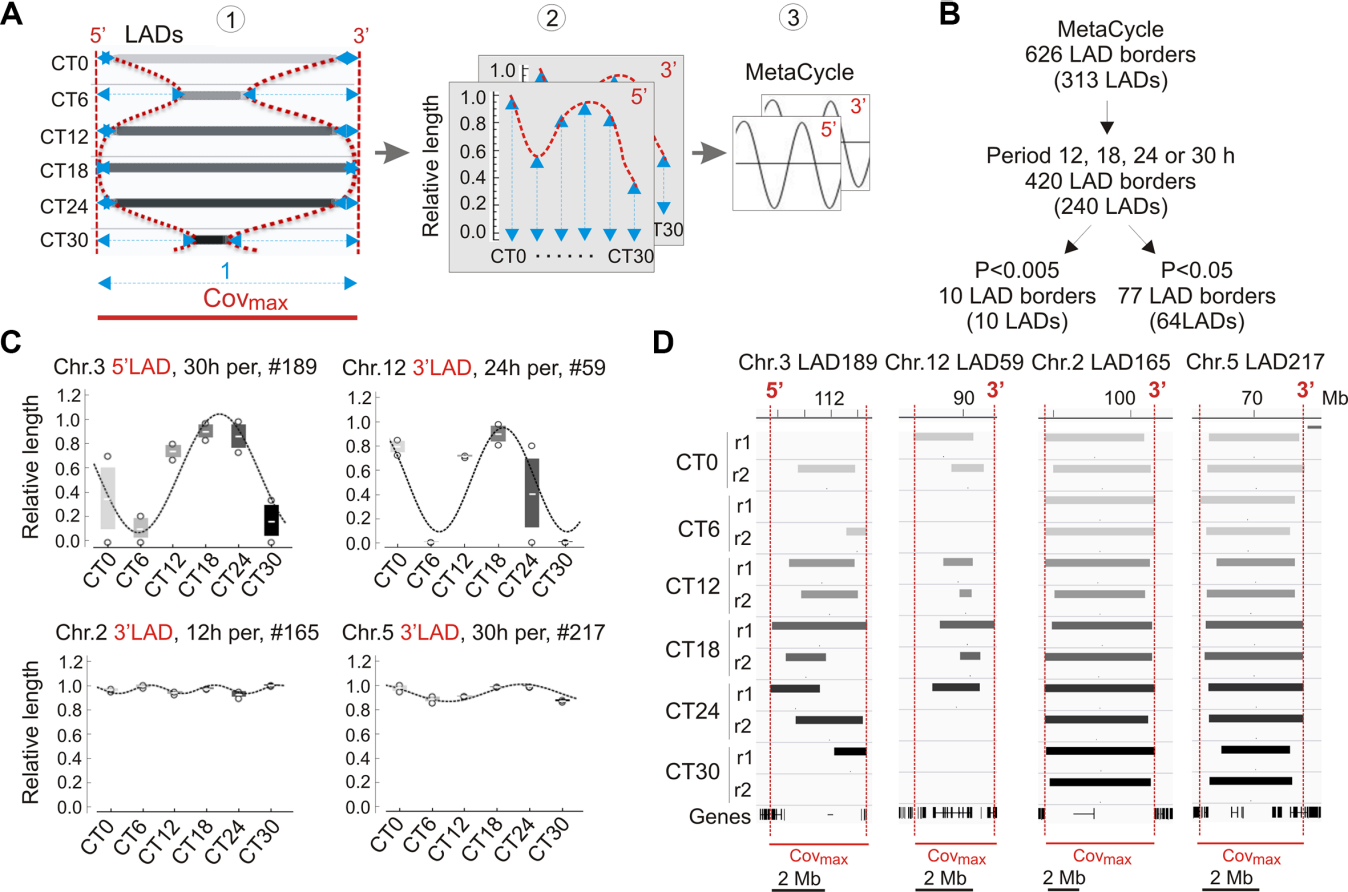
Analysis of periodicity in genome coverage by LADs. **(A)** Approach to the identification of periodic LADs. (1) A variable LAD area is identified, for each LAD, across CTs and replicates (not shown here for clarity); the maximal merged area of these LADs is defined as Cov_max_ and distances from the 5′ and 3′ end of each LAD to the Cov_max_ limits are measured (blue arrows); (2) relative lengths are calculated at both the 5′ and 3′ end of LADs (1 = Cov_max_ length; 0 = no LAD); (3) MetaCycle is applied to identify periods at the 5′ and 3′ ends of LADs. **(B)** Identification of periodic LADs using MetaCycle. **(C)** Examples of periodic oscillations (*P* < 0.005; MetaCycle Fisher’s exact test) in LAD 5′ and 3′ length during the circadian cycle among the 10 LADs identified by MetaCycle (see also **Table 1**); mean ± SD, individual data points and MetaCycle best-fit cosine curves are shown. **(D)** Genome browser views of periodic LADs shown in **(C)**. Red lines delimit Cov_max_ and the 5′/3′ numbering denotes the periodic LAD border shown in **(C)**.

We next examined variations in LADs more closely during the circadian cycle. These occur by LAD extension or shortening, sometimes resulting in a fusion or splitting of LADs, or by formation and dissociation of entire LADs (see **Figures 3A and B**). We therefore devised a strategy to quantitatively characterize the genomic coverage of these variable LADs over time. We determined for each LAD the maximal genome coverage (cov_max_) in the CT0–CT30 time course as the union of LAD coverage across all replicates and CTs, and ascribed to cov_max_ a reference value of 1 (**Figure 4A**, left panel). The 5′ and 3′ boundaries of this cov_max_ area provided genomic coordinates for measures of variations in LAD length within this area. For each CT and replicate, variable 5′ and 3′ LAD lengths were extracted and standardized from 0 to 1 (cov_max_), 0 referring to the complete disappearance of a LAD, and 1 corresponding to the entire LAD being present over the whole cov_max_ area (**Figure 4A**, middle panel). We then used MetaCycle to identify any periodicity in the extension or shortening of LADs within the cov_max_ area (**Figure 4A**, right panel), where MetaCycle applied a cosine curve best-fitting genome coverage variations at the 5′ and 3′ end of LADs.

We applied MetaCycle to 626 variable LAD borders (at 313 LADs) to identify periodically oscillating lengths of these LADs over the CT0–CT30 time course (**Figure 4B**). Among these, MetaCycle tentatively identifies 5′ and/or 3′ end oscillations with 12, 18, 24, or 30 h (± 3 h) periods in 420 LAD borders (among 240 LADs; **Figure 4B**; **Supplementary Table S8**). Ascribing a stringent *P* value of < 0.005 (Fisher’s exact test; as in our MetaCycle RNA-seq analysis) returns 10 highly significantly periodic LADs with periods distributed throughout the circadian cycle (**Figure 4B**; **Table 1**; **Supplementary Table S8**). Relaxing the *P* value to *P* < 0.05 expectedly increases the number of periodic LAD borders to 77 (30 5′-periodic, 47 3′-periodic, and 13 both 5′- and 3′-periodic) among 64 distinct LADs (**Figure 4B**; **Supplementary Table S8**). However, inspection of the profiles of these LADs revealed, for some of them, discrepancy with the best curve fitted by MetaCycle. This led us to focus our subsequent analysis on the 10 LADs identified above at *P* < 0.005, which we henceforth refer to as “periodic LADs” (**Figures 4C and D**). Periodic LADs therefore constitute a subset of variable LADs with highly significant periodic oscillations in the genomic coverage in their 5′ or 3′ ends. These LADs withstand a randomization test of all replicates and CTs (see Materials and Methods section), suggesting that LAD periodicity observed in our data is imposed by the order of CTs. Periodic LADs include LADs with a stable core and variable borders, and LADs that entirely appear or disappear (**Figures 4C and D**). Altogether, our data indicate that significant periodicity in LAD border interactions with LMNB1 only concerns a minor set of LADs. Thus, the majority of LMNB1–chromatin interactions are conserved during the circadian cycle.

**Table 1:** Period of 5′ and 3′ significantly periodic LAD borders.

LAD no.	Chr.	5′ period (h)	3′ period (h)
15	1	–	12
165	2	–	12
167	2	24	–
189	3	30	–
194	3	–	12
217	5	–	30
239	7	–	18
262	8	12	–
36	10	18	–
59	12	24	–

### Periodic Gene Expression Is Uncoupled From Rhythmic LMNB1–Chromatin Interactions

**Figure 5:**
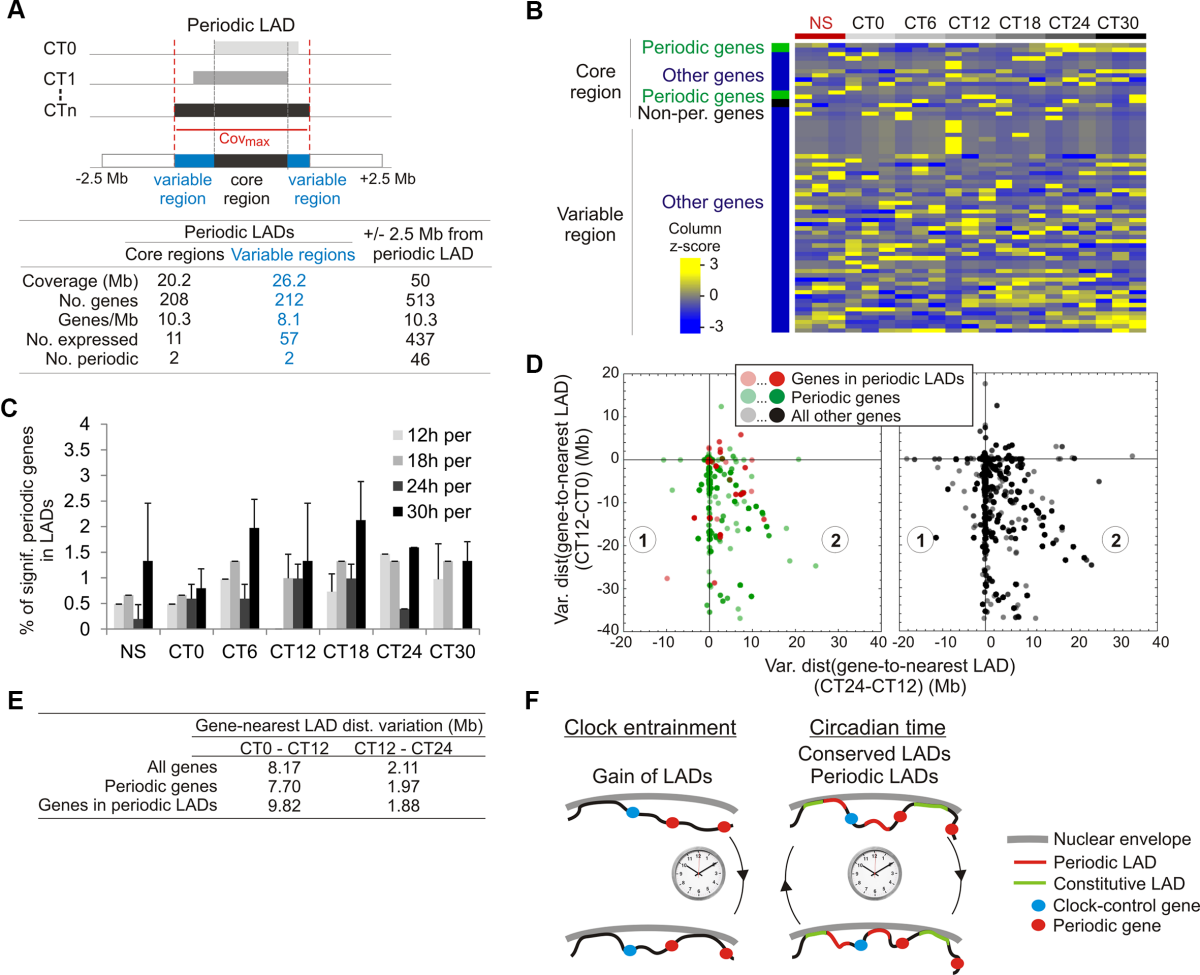
Genes in or near periodic LADs do not display periodic expression. **(A)** Representation and gene statistics of the core and variable regions of the significantly periodic LADs. **(B)** Gene expression z-score of expressed genes (n = 63) localized in significantly periodic LADs, for each CT and replicate (n = 3). Genes are sorted by groups (indicated on the left) and within groups, by phase. **(C)** Percentages of significantly periodic genes (*P* < 0.005, MetaCycle Fisher’s exact test) found in LADs at each CT. **(D)** Scatter plots of variations in gene-to-nearest LAD distances between CT0 and CT12 (y axis) and between CT12 and CT24 (x axis). One data-point represents a single gene or multiple genes at a particular coordinate on the graph, with color intensity reflecting the number of genes at that point. **(E)** Mean gene-to-nearest LAD distance variation. **(F)** Summary and model of oscillatory LAD patterns after entrainment of the circadian clock. Entrainment of the clock is accompanied by a net gain of LMNB1 LADs (bottom). During circadian time, most LADs are conserved (constitutive LADs) while a small number is periodic. Typical genomic positions of clock-control genes and periodic genes relative to these LADs are shown.

We then examined the relationship between periodic LADs and gene expression. Out of 430 genes found in periodic LADs, only 68 genes are expressed, albeit with no periodicity (**Figures 5A and B**; **Supplementary Figure S4A**). Moreover, we find that the vast majority of periodic genes are outside LADs at any time point during the circadian cycle (only < 2% are in LADs; **Figure 5C**). We then examined genes located within 2.5 Mb of the 5′ or 3′ end of periodic LADs (523 genes). Most of these genes are expressed (437genes), among which ∼10% are periodic, yet again with no dominant period and no temporal relationship to LAD periodicity (**Figure 5A**). Thus, since most genes reside outside LADs during the circadian cycle and no specific feature was identified in periodic LADs, we then determined at each CT the relationship between genes, and in particular periodic genes, and gene distance to the nearest LAD (see Materials and Methods). We find that the minimal distance occurred at CT12 while the maximal distance was detected at CT0 and CT24/30 (**Supplementary Figure S4B**). We thus examined the magnitude of variations in gene-to-nearest-LAD distance occurring between CT0 and CT12 and between CT12 and CT24 (to maintain 12-h intervals in both cases during a 24 h circadian time). Consistent with this transient gain in LAD coverage (see **Figure 3E**) and with the gene-poor content of LADs, we observe an overall decrease in gene-to-nearest LAD distance between CT0 and CT12 (**Figure 5D**; data points in quadrants 1 and 2 and along the y axis, negative values), and for most genes, an increase thereafter, between CT12 and CT24 (data points in quadrant 2). These changes concern both periodic genes (*left panel*, green data points) and all other genes (*right panel*) regardless of the magnitude of this variation (**Figure 5D**; y axis negative values). Of note, the magnitude of this variation (**Figure 5E**) is larger than that of the intrinsic cumulated EDD error at each CT involved (see **Supplementary Table S2**, EDD error) and larger than the LAD variation between LAD intersects and replicates (see **Supplementary Table S7**, LAD variation).

We conclude that there is no correlation between periodicity in gene expression and variation in gene-to-nearest LAD distance. This variation appears to be caused by a transient gain in the number of LADs, the functional significance of which remains to be examined. Similarly, the central clock-regulating genes are typically megabases away from the nearest LAD (**Supplementary Table S9**; **Supplementary Figure S4C**), and promoter regions of these genes are also essentially devoid of LMNB1 interaction at any CT (**Supplementary Figure S4D**).

Altogether, our results argue for a constitutive localization of circadian genes, including clock regulators, in a chromatin environment essentially devoid of B-type lamin interactions, providing permissiveness for periodic transcriptional activation during the circadian cycle (Koike et al., 2012; Kim et al., 2018). Our findings argue that oscillatory expression of periodic genes, including central clock-control genes, is uncoupled from a direct association with the nuclear lamina or from their localization in the vicinity of periodic LADs.

## Discussion

Oscillations in chromatin conformation mediated by rhythmic chromosomal interactions contribute to the regulation of circadian gene expression (Aguilar-Arnal et al., 2013; Xu et al., 2016; Kim et al., 2018; Mermet et al., 2018; Yeung et al., 2018). We now provide evidence of a net gain of lamin–chromatin interactions following entrainment of the circadian clock (NS-CT0 transition), which speculatively may reinforce the robustness of segregation of chromatin domains in the nucleus space.

Given the overall repressive chromatin environment at the nuclear periphery (van Steensel and Belmont, 2017), and evidence of cyclic recruitment and silencing of specific genes at the nuclear envelope in a human cancer cell line (Zhao et al., 2015), we reasoned that periodic gene expression could at least in part be regulated by periodic interactions with the nuclear lamina. We applied MetaCycle, a tool designed to identify periodic transcript oscillations (Wu et al., 2016), to identify periodic changes in genome coverage by LMNB1. Our approach distinguishes 5′ and 3′ LAD extension or shortening from entire LAD emergence or disappearance (**Figure 5F**). The gain of lamin–chromatin interactions between NS to CT0 is followed by periodic interactions of specific genomic regions with LMNB1, suggestive of discrete rhythmic associations with the nuclear lamina. Our ChIP approach would notably detect interactions of chromatin with nucleoplasmic LMNB1, which appear to be more euchromatic than peripheral LADs (Pascual-Reguant et al., 2018), suggesting that periodic LADs may also be nucleoplasmic. The detection of periodic LADs in our study adds to mounting evidence that chromatin is able to display oscillations in its 3-dimensional conformation (Kim et al., 2018).

Counterintuitively however, several points in our data argue that in the mouse liver, periodic gene expression is not directly coupled to oscillating or periodic chromatin associations with the nuclear lamina. (1) Genes with periodic transcript levels reside outside LADs at any time point during the circadian cycle. This notably includes *Pard3*, which has been found to be periodically recruited to the nuclear envelope in human colon cancer cells (Zhao et al., 2015), but in mouse liver is localized 15 Mb away from the nearest (and conserved) LAD. (2) Similarly, our analysis reveals that central clock-control genes reside megabases away from the nearest LAD and show no promoter association with LMNB1. This configuration may keep these genes in a transcriptionally permissive (“lamin-free”) environment throughout the circadian cycle, compatible with a regulation of circadian transcription by rhythmic TF binding and activity (Koike et al., 2012; Masri et al., 2014; Zhang et al., 2015). Our results also suggest that rhythmic chromatin looping activity which may regulate gene expression within TADs (Kim et al., 2018) take place in an environment where chromatin is not restrained by nuclear lamins (Bronshtein et al., 2015). (3) Periodic genes can also flank constitutive LADs and conversely, genes with stable (high or low) expression levels may flank periodic LADs. (4) Lastly, there is no relationship between periodic gene expression and gene-nearest LAD distance during the circadian cycle. We find that periodic genes are primarily involved in chromatin regulation, transcription regulation and several metabolic functions. Our data strongly suggest therefore that the periodic LAD patterns identified here in liver cannot readily explain these oscillatory gene expression patterns. In fact, periodic hepatic gene expression as a whole appears to be uncoupled from periodic chromatin association with the nuclear lamina.

Concurring with our results, other chromatin-linked processes are uncoupled from gene expression patterns. For example, many oscillatory genes display stable chromosomal interactions (Kim et al., 2018), including promoter–enhancer contacts (Beytebiere et al., 2019) during the circadian cycle. Conversely, many expressed genes display circadian oscillations in promoter and enhancer histone modification patterns that are irrespective of whether or not these genes are periodic or not (Koike et al., 2012). These observations highlight a complex and in some instances enigmatic cross-talk between circadian transcription and rhythmic changes in chromatin states.

Our findings raise several important issues. First, periodic LADs are not a prominent feature of LMNB1–chromatin interactions during the circadian cycle. Sixty-four LADs display variations in their 5′ and/or 3′ end coverage, but we only find, using our stringent approach, ten highly significantly periodic LADs, with asynchronous oscillations between their 5′ and 3′ borders. Yet, these variations withstand a randomization test, suggesting that rhythmicity is elicited by the underlying order of CTs (i.e. circadian time) rather than by random lamin–chromatin interactions. Our findings suggest therefore that the periodic LMNB1–chromatin interactions identified here represent a small subset of all variable interactions. Nevertheless, although we find no evidence of punctual interactions of LMNB1 with individual clock-control genes or promoters, it remains possible that a subset of genes, including periodic genes, display discrete circadian interactions with LMNB1 reminiscent of those shown in cancer cells (Zhao et al., 2015). In addition, periodic LADs do not seem to harbor any evident functional properties. This contrasts with developmentally regulated B-type lamin–chromatin interactions which have been reported during differentiation of mouse embryonic stem cells (Peric-Hupkes et al., 2010). On the other hand, differential LMNB1 LADs have also been reported in hepatocarcinoma cells treated with cyclosporin, albeit with no significant changes in gene expression (Forsberg et al., 2019), akin to what we observe in the liver. It remains to be examined whether LADs, and periodic LADs in particular, described here involve a level of regulation which currently remains unappreciated.

Second, how could periodic lamin–chromatin interactions be regulated? The cistrome of circadian genes can oscillate in a manner concordant with circadian gene expression (Feng et al., 2011; Fang et al., 2014; Zhang et al., 2015), thus factors mediating lamin–chromatin interactions may also be periodically recruited to target loci. Our transcriptome data indicate that several genes encoding BTB/POZ domain proteins oscillate during the circadian cycle. These proteins share DNA binding motifs enriched in LADs (Guelen et al., 2008) and in lamin-associated sequences (Zullo et al., 2012; Lund et al., 2013), and are found in sequences able to re-localize chromatin to the nuclear lamina (Harr et al., 2015). Thus, oscillating LADs could potentially be regulated through the periodic recruitment of factors important for chromatin localization at the nuclear periphery (Shachar et al., 2015). A search for protein binding motifs in the core and variable regions of periodic LADs using several tools including HOMER (Heinz et al., 2010) and the MEME suite (Bailey et al., 2009), however, revealed no significant enrichment in known motifs which could point to protein candidates mediating these periodic lamin–chromatin interactions. Speculatively, proteins of the inner nuclear membrane or bound to the nuclear lamina, through rhythmic post-translational modifications or through their periodic recruitment to these nuclear domains, could also mediate periodic interactions by direct or indirect interactions with chromatin. A discovery of the proteome of the nuclear periphery or of interactome of nuclear lamins during the circadian cycle would be valuable in the identification of periodic association of chromatin with the nuclear lamina.

Third, what would be the significance of oscillating, or taken more broadly, variable lamin–chromatin interactions and their impact on genome architecture? Resetting of LADs immediately after entrainment of the circadian clock may strengthen the robustness of liver-specific gene expression, possibly through a marked segregation of heterochromatin from euchromatin (Solovei et al., 2013; Falk et al., 2019). Oscillations of subsets of LADs, regardless of periodicity, may alter the radial positioning of chromatin and/or confer dynamic changes in chromatin states in regions that are in 3-dimensional proximity, but not necessarily in linear proximity. These changes altogether may affect gene expression in some of these regions (Robson et al., 2016; Paulsen et al., 2019), but not necessarily in all (Forsberg et al., 2019). LAD displacement may also result in radial repositioning of topological chromatin domains (Robson et al., 2016), or of regulatory elements (Robson et al., 2017). Assessment of periodic LADs in a 3-dimensional context should shed light on the putative long-range impact of LAD dynamics on genome architecture and function.

## Data Availability Statement

Lamin B1 ChIP-seq and RNA-seq data have been deposited to the NCBI GEO database and are available under GEO accession No. GSE128675.

## Ethics Statement

Animal procedures were approved by the Norwegian Regulatory Authorities (Approval No. 8565).

## Author Contributions

FF, AB and PC designed the study. FF and QF did wet-lab experiments. TS supervised parts of these experiments. AB did the bioinformatics analyses. AB and PC supervised the work and wrote the manuscript.

## Conflict of Interest

The authors declare that the research was conducted in the absence of any commercial or financial relationships that could be construed as a potential conflict of interest.

## Abbreviations

ChIP-seq, chromatin immunoprecipitation sequencing; CT, circadian time; LAD, lamina-associated domain; NS, non-synchronized; PBS, phosphate buffered saline; PMSF, phenylmethylsulfonyl fluoride; RNA-seq, RNA-sequencing; RT-qPCR, reverse transcription quantitative polymerase chain reaction; TF, transcription factor.
