# Supplementary material for: Nuclear Lamin B1 Interactions With Chromatin During the Circadian Cycle Are Uncoupled From Periodic Gene Expression
Source: Front Genet. 2019 Oct 3;10:917. doi: 10.3389/fgene.2019.00917 (PMC6785633; doi:10.3389/fgene.2019.00917)
Supplement: Supplementary file 1 [file Presentation_1.pdf]

Brunet, Forsberg et al.

Supplementary information

Supplementary Table S1. RT-qPCR and ChIP-qPCR primers used in this study.
Supplementary Table S2. LAD detection parameters using EDD.
Supplementary Table S3. MetaCycle analysis of expression of central clock regulators and *Lmnb1* determined by RT-qPCR and RNA-seq.
Supplementary Table S4. Lists of periodic and non-periodic genes. (Excel; classifiable lists-Supplementary Table 4)
Supplementary Table S5. Periodic genes classified by phase within periods. (Excel - Supplementary Table 5)
Supplementary Table S6. Gene ontology terms enriched for periodic genes.
Supplementary Table S7. Analysis of LAD intersects.
Supplementary Table S8. Characterization of periodic LADs by MetaCycle. (Excel; classifiable lists - Supplementary Table 8)
Supplementary Table S9. Distances between central clock-regulating genes and nearest LADs.

Supplementary Table S1. RT-qPCR and ChIP-qPCR primers used in this study.

Amplicon	Chr.	Forward primer (F)<br>Reverse primer (R)	mm10<br>Genomic position (nt)
**RT-qPCR primers**			
Clock	11	F: CCTAGAAAATCTGGCAAAATGTCA<br>R: CCTTTTCCATATTGCATTAAGTGCT	
Per1	1	F: ACCAGCGTGTCATGATGACATA<br>R: GTGCACAGCACCCAGTTCCC	
Arntl	5	F: GCAGTGCCACTGACTACCAAGA<br>R: TCCTGGACATTGCATTGCAT	
Nr1d1	2	F: CTGGAGGGCTGCAGTATAGC<br>R: CGGTCATTCAAACTGGACCT	
Cry1	16	F: CAGACTCACTCACTCAAGCAAGG<br>R: TCAGTTACTGCTCTGCCGCTGGAC	
Eif2a	3	F: CAACGTGGCAGCCTTACA<br>R: TTTCATGTCATAAAGTTGTAGGTTAGG	
**ChIP-qPCR primers**			
cLAD-1 chr.11	11	F: TAACCACCGTGTCTCTAAGCG<br>R: TCCGGTGTGACTTAGCATGA	chr11:81201226-81201309
cLAD-1 chr.1	1	F: GCCACCAACATGGAGTCAGA<br>R: CAGGCTCTGAATGGAGCCAA	chr1:190487280-190487361
cLAD-1 chr.5	5	F: TGCTGTGAGCATTTGGTCCT<br>R: GCCTCCACCTCAGTCACTTC	chr5:126224330-126224438
cLAD-1 chr.2	2	F:CCTGACTTTTACCGTCCCAATC<br>R: TGCTACTACACAAGGCCCCAA	chr2:156231214-156231331
cLAD-1 chr.16	16	F: GTTTTTCATCGGACGGTGGC<br>R: CCGCCGCTCATACACCATAA	chr16:10796381-10796520
cLAD-2 chr.1	1	F: CCACAGCATCCCAGTTTCCT<br>R: CAGGGACCTTCCAGGTTTCC	chr1:113441259-113441360
cLAD-2 chr.5	5	F: TCTCTGCCACAGACTGCAAG<br>R: GCCTTCTGGTCTCCTGCTTT	chr5:55584684-55584784
cLAD-1 chr.12	12	F: AGCTCCTTGTCTTGCCTGTC<br>R: AGAATAGCGAGTGCCCAACC	chr12:47158301-47158371
cLAD-2 chr.16	16	F: TGGAGTTCTGCAAGGCTGAG<br>R: ACTGCTTGCTGCTCTCCTTT	chr16:82996670-82996796
vLAD chr.1	1	F: GGTGGCATCAGCTCCAAAGT<br>R: AGCTTTTATTTTCTCAAAGGCAGGT	chr1:123423631-123423729
vLAD chr.5	5	F: CAGTTGCTGGGACTGTAGCA<br>R: AAAGTGTGAGCCCAGGATGG	chr5:48143370-48143444
vLAD chr.12	12	F: GAGGAGCAAACCTGTTCCCA<br>R: AGCTTTTACTCTGTGCCCCC	chr12:38104881-38105004
vLAD chr.16	16	F: TCCCCAGCAACATTCCAGAC<br>R: AGCAACAAATGTGCAGGCAG	chr16:39947233-39947346
nonLAD chr.6<br>*(Gadh* promoter)	6	F: GTTGCACTGGCCTAGCAAAG<br>R: GACCGGGATTCTTCACTCCG	chr6:125166811-125166927
nonLAD chr.7	7	F: CACCGGCTTCACCGAATACT<br>R: GGAGAGAAACGGGGTTCCG	chr7:25315585-25315719
nonLAD chr.11<br>(*Ube2b* promoter)	11	F: GCCGAACTTGAAACTAGCGAC<br>R: GTTGCTTGCGGGCAACTACG	chr11:52000592-52000714
nonLAD chr.18<br>*(Lmnb1* promoter)	18	F: GGTCAAGTCATTGGTGGGCT<br>R: TCATTGGCTATGAACGCGGA	chr18:56707121-56707212

cLAD, constitutive LAD; vLAD, variable LAD.

Supplementary Table S2. LAD detection parameters using EDD.

		NS		T0		T6		T12		T18		T24		T30	
		Rep1	Rep2	Rep1	Rep2	Rep1	Rep2	Rep1	Rep2	Rep1	Rep2	Rep1	Rep2	Rep1	Rep2
EDD parameters	Mean(BinSize) (bp)	61000	4000	47000	4000	35000	11000	1000	5000	19000	6000	14000	18000	31000	46000
	Mean(GapPenalty)	3.70	6.20	3.50	5.00	4.40	4.30	4.10	6.40	3.60	3.60	3.50	4.20	4.00	5.00
	StdDev(GapPenalty)	1.20	0.50	0.40	0.20	0.80	0.50	0.40	0.10	0.40	0.10	0.30	0.30	0.80	1.60
	Edd Error (Mb)	0.98	1.24	0.11	0.16	0.95	0.30	0.11	0.12	0.46	0.19	0.34	0.51	0.78	0.69
# RemovedLADs		0	79	0	39	0	4	4	39	0	13	0	0	0	0
# LADs in replicates		74	372	97	320	130	280	242	300	154	346	179	183	138	127
# LADs in intersect		93		111		153		225		198		163		111	

Supplementary Table S3. MetaCycle analysis of expression of central clock regulators and *Lmnb1* determined by RT-qPCR and RNA-seq.

	Gene	P-value ID	P-value	Period ID	Period	Phase	Base	Amplitude
RT-qPCR	*Lmnb1*	Non-Significant	9.8E-01	12h	13.7	13.2	0.3	0.0
	Clock	Periodic	4.7E-04	24h	23.7	23.6	0.9	0.6
	Arntl	Periodic	3.1E-10	24h	23.1	22.3	0.5	0.5
	Cry1	Periodic	1.7E-06	24h	22.7	19.7	3.9	3.5
	Per1	Non-Significant	7.2E-02	18h	15.0	13.7	0.3	0.4
	Nr1d1	Periodic	5.1E-11	24h	23.8	7.9	0.9	1.1
RNA-seq	*Lmnb1*	Non-Significant	1.6E-01	12h	12.0	6.7	6.5	0.6
	Clock	Periodic	4.6E-06	24h	22.8	1.0	17.3	4.7
	Arntl	Periodic	2.1E-06	24h	23.0	0.0	17.0	14.2
	Cry1	Periodic	1.2E-08	24h	23.0	19.9	23.1	14.3
	Cry2	Non-Significant	1.4E-02	18h	18.0	9.6	26.4	1.6
	Per1	Non-Significant	2.1E-01	18h	18.7	13.6	14.1	11.9
	Per2	Periodic	2.7E-07	30h	29.3	14.3	17.7	11.3
	Per3	Periodic	3.0E-06	24h	23.9	14.1	13.1	10.1
	Nr1d1	Periodic	7.1E-06	24h	24.4	8.2	41.7	39.7
	Nr1d2	Periodic	1.7E-03	24h	24.2	11.3	24.2	10.6
	Rorc	Periodic	3.2E-05	30h	28.0	19.7	50.6	19.9
	Ube2b	Non-Significant	1.3E-01	24h	21.1	15.8	43.6	0.4

For MetaCycle analysis, see Main text; these values are used for best-fits. Function used for best-fit:
$F[x] = Amplitude*Cos[(2\pi/Period)\ (x - Phase)] + Base$.

Supplementary Table S6. Gene ontology terms enriched for periodic genes.

PANTHER GO-Slim Biological Process	No. genes in ref. genome	No. genes in GO term	Expected No. genes	Fold Enrichment	P-value	FDR	Over/under representation
**12h period**							
DNA repair	153	8	1.4	5.73	1.12E-04	9.08E-03	+
regulation of biological process	3320	12	30.27	0.4	9.70E-05	1.18E-02	-
nitrogen compound metabolic process	2462	42	22.45	1.87	7.02E-05	1.71E-02	+
nucleobase-containing metabolic process	2710	42	24.71	1.7	5.41E-04	2.20E-02	+
cellular component biogenesis	733	17	6.68	2.54	4.89E-04	2.39E-02	+
RNA metabolic process	1536	28	14.01	2	4.64E-04	2.83E-02	+
**18h period**							
None							
**24h period**							
rhythmic process	13	4	0.15	27.4	3.15E-05	2.57E-03	+
circadian rhythm	13	4	0.15	27.4	3.15E-05	3.85E-03	+
metabolic process	6009	98	67.48	1.45	3.05E-05	7.44E-03	+
sensory perception of smell	699	0	7.85	< 0.01	6.84E-04	2.78E-02	-
sensory perception of chemical stimulus	748	0	8.4	< 0.01	4.84E-04	2.95E-02	-
G-protein coupled receptor signaling pathway	875	1	9.83	0.1	8.52E-04	2.97E-02	-
protein localization	494	15	5.55	2.7	6.09E-04	2.97E-02	+
**30h period**							
DNA binding transcription factor activity	34	5	0.27	18.29	1.41E-05	3.44E-03	+
regulation of molecular function	436	12	3.51	3.42	2.67E-04	3.25E-02	+
**Non-periodic**							
None							

+, over-representation; -, under-representation.

Supplementary Table S7. Analysis of LAD intersects.

	NS		T0		T6		T12		T18		T24		T30	
	Rep1	Rep2	Rep1	Rep2	Rep1	Rep2	Rep1	Rep2	Rep1	Rep2	Rep1	Rep2	Rep1	Rep2
	vs. Intersect	vs. Intersect	vs. Intersect	vs. Intersect	vs. Intersect	vs. Intersect	vs. Intersect	vs. Intersect	vs. Intersect	vs. Intersect	vs. Intersect	vs. Intersect	vs. Intersect	vs. Intersect
LAD size variation (Mb)	001	001	001	001	000	001	000	000	000	001	001	001	001	001
LAD coverage per replicate (Mb)	335.29	526.51	423.75	574.97	495.17	584.04	586.34	553.19	617.87	662.99	631.35	540.44	405.39	392.37
LAD coverage (intersect) (Mb)	258.87		339.59		434.22		496.13		515.81		474.77		311.99	

Supplementary Table S9. Distance between central clock-regulating genes and nearest LADs.

Gene	Chr.	Dist. 5' end of gene to LAD (Mb)	Dist. 3' end of gene to LAD (Mb)
Clock	5	-4.2	+1.6
Arntl (Bmal1)	7	-5.2	no LAD
Cry1	10	-10.8	+12.6
Per1	11	-20.8	+21.6
Per2	1	-41.4	+2.7
Per3	4	-3.2	no LAD
Nr1d1	11	-5.3	no LAD
Nr1d2	14	-10.5	+19.3
Rorc	3	-1.5	+15.7

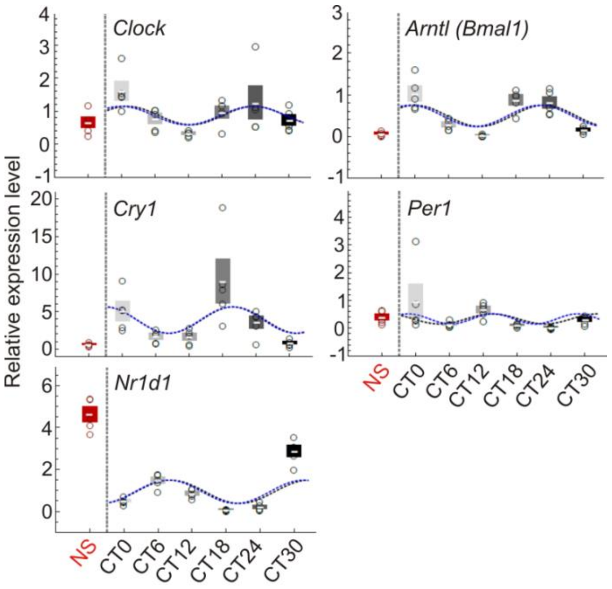

Supplementary Figure S1. Circadian expression of central clock genes. RT-qPCR analysis of expression of clock genes in non-synchronized (NS) mice and from CT0 to CT30 (mean ± SD, single data points; n = 5 mice at NS and per CT). Blue line, MetaCycle best-fit from RT-qPCR data; black line, MetaCycle best-fit from RNA-seq data; note the strong overlap between the two. Amplitude and base values used for both fits are from RT-qPCR MetaCycle analysis.

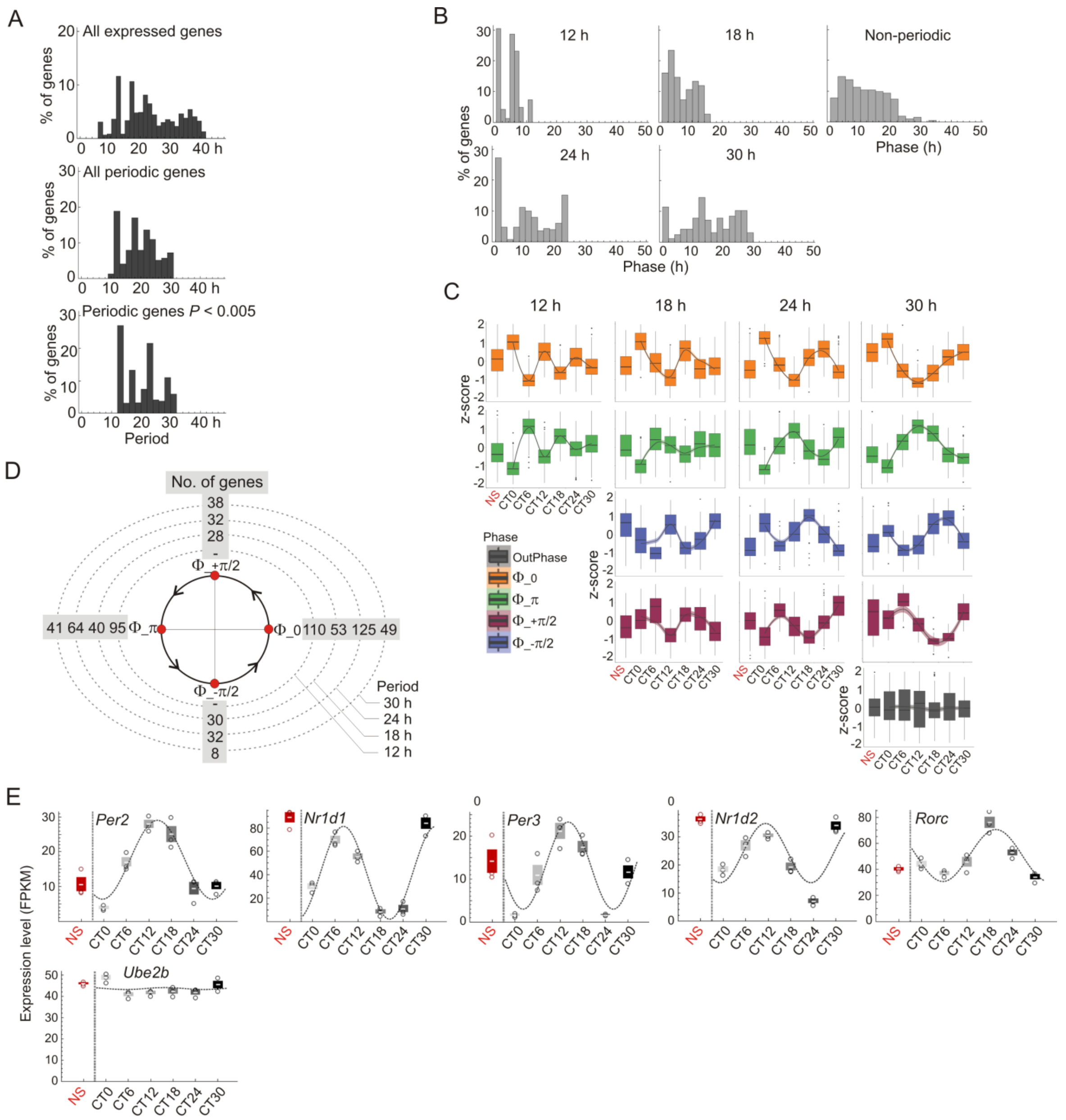

Supplementary Figure S2. Characterization of periodic gene expression in liver. **(A)** Period distribution of all 17,330 expressed genes, all periodic genes and significantly ($P < 0.005$; Fisher's exact test) periodic genes. **(B)** Phase distribution of periodic genes (x axis), shown as % of genes in indicated periods. **(C)** Expression of periodic genes according to their phase (median, upper/lower quartiles, maxima/minima and smoothing of medians ± SD). **(D)** Trigonometric clock and number of significantly ($P < 0.005$) periodic genes with indicated period (circle dotted lines) and phase (central circular axis). **(E)** RNA-seq analysis of circadian expression of central clock genes and the housekeeping gene *Ube2b* (n = 3 mice per CT; mean ± SD, individual data points). Cosine curves in are best-fits from MetaCycle; fitting function F[x] = Amplitude*Cos[(2π/Period) (x - Phase)] + Base; see **Supplementary Table S3**.

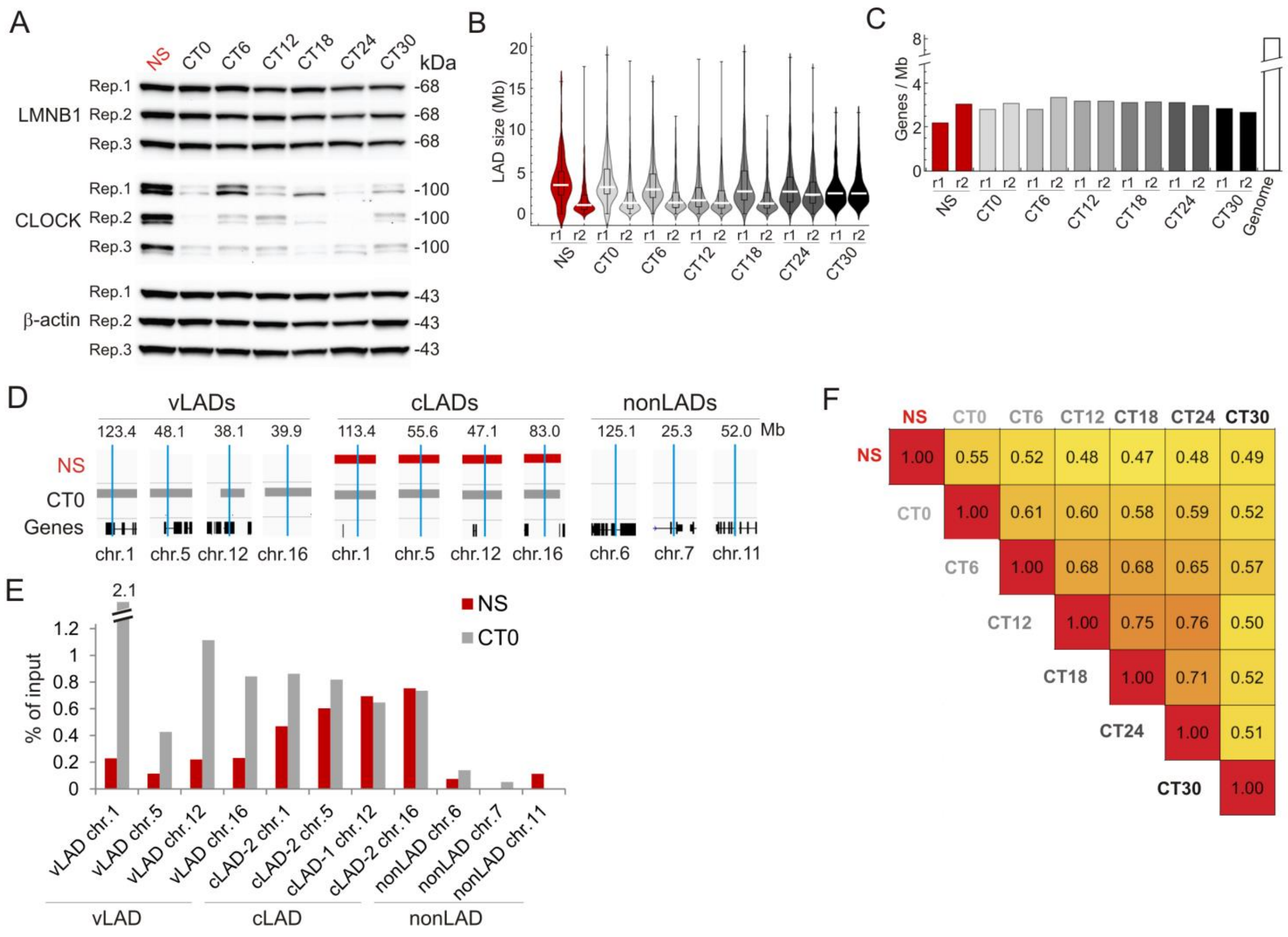

Supplementary Figure S3. Characterization of LMNB1 LADs during the circadian cycle. **(A)** Western blots of LMNB1 and CLOCK expression; β-actin was used as loading control (biological triplicate). Each replicate blot (Rep.1/2/3) is from the liver of one mouse per CT. **(B)** Median (white bars) and violin plot distribution of LAD sizes for each CT and biological replicate (r1, r2). **(C)** Gene density in LADs and in the mm10 genome. **(D)** Genome browser views of variable LAD (vLAD), constitutive LAD (cLAD) and nonLAD regions at NS and CT0, with position of amplicons (blue bars, in Mb on indicated chromosomes; bottom) analyzed by ChIP-qPCR in (E). **(E)** ChIP-qPCR analysis of LMNB1 enrichment in indicated vLAD, cLAD and nonLAD regions; see **Supplementary Table S1** for position of amplicons. **(F)** Jaccard indices of LAD coverage overlap between CTs (values are for for LAD intersects of both replicates at each CT).

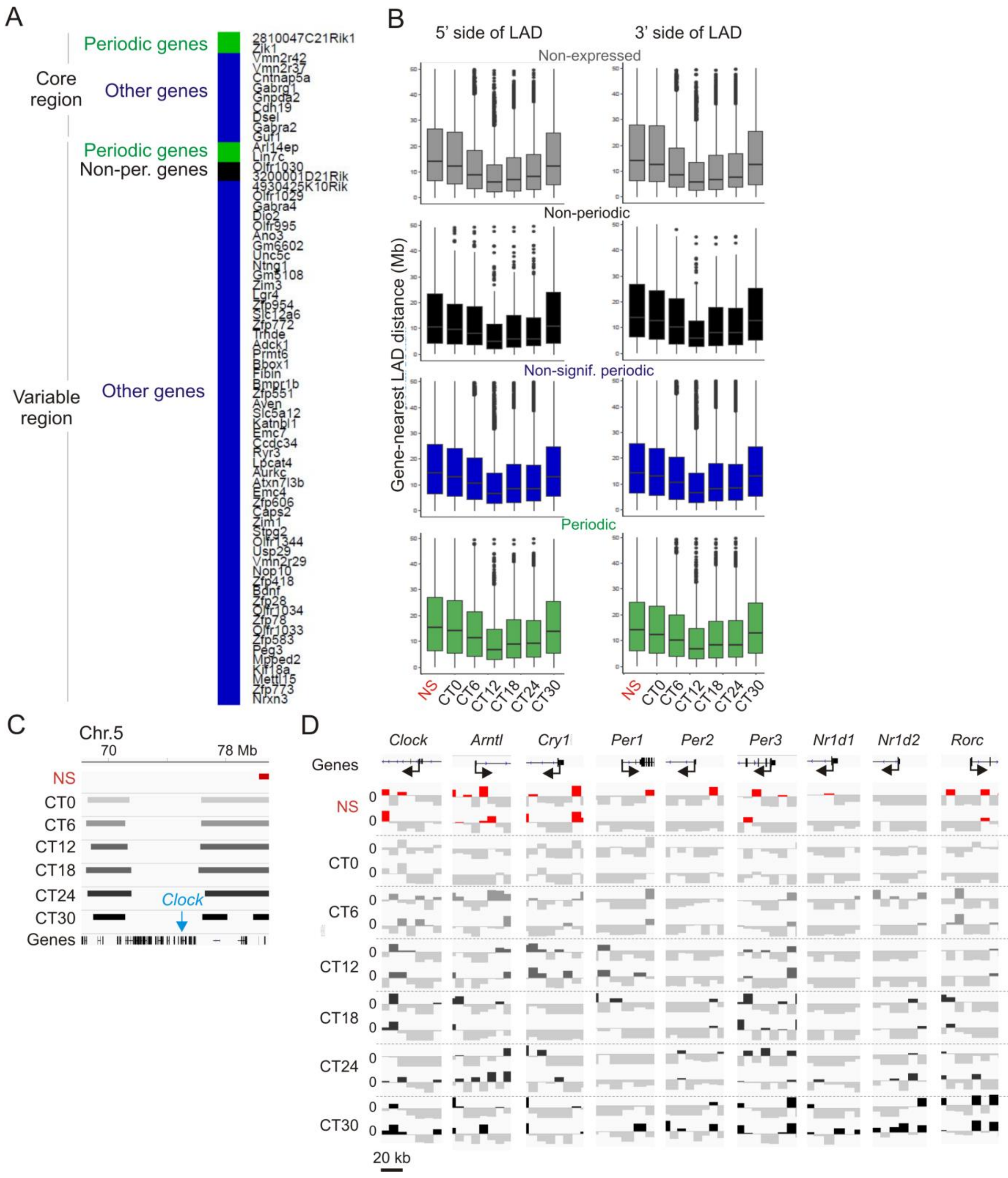

Supplementary Figure S4. Gene-to-nearest LAD distances are uncoupled from periodic gene expression. **(A)** List of genes in periodic LADs, classified in the core and variable regions in **Figure 5B**. **(B)** Box plots of variation in gene-to-nearest LAD distance at the 5' and 3' sides of LADs (median, upper/lower quartiles, maxima/minima); data are shown for non-expressed genes (n = 4820), non-periodic genes (n = 204), non-significantly periodic genes (n = 15685) and periodic genes (n = 1441). **(C)** Genome browser view of LADs in a fragment of chromosome 5 showing *Clock* between two LADs. **(D)** The transcription start site (arrow) of central clock-control genes is devoid of LMNB1 interaction detectable by ChIP. The panel shows genome browser views of LMNB1 enrichment in promoter regions for each ChIP replicate and CT. Data are shown for 10 kb bins, as log2(ChIP/input) ratios (y axis, scale: -0.3 to +0.3).
